# Ultrasensitive all-2D MoS_2_ phototransistors enabled by an out-of-plane MoS_2_ PN homojunction

**DOI:** 10.1038/s41467-017-00722-1

**Published:** 2017-09-18

**Authors:** Nengjie Huo, Gerasimos Konstantatos

**Affiliations:** 1 ICFO—Institut de Ciencies Fotoniques, The Barcelona Institute of Science and Technology, Castelldefels, 08860 Barcelona, Spain; 2 ICREA—Institució Catalana de Recerca i Estudis Avançats, Lluis Companys 23, 08010 Barcelona, Spain

## Abstract

Two-dimensional transition metal dichalcogenide-based photodetectors have demonstrated potential for the next generation of 2-dimensional optoelectronics. However, to date, their sensitivity has not been superior to that of other technologies. Here we report an ultrasensitive two-dimensional photodetector employing an in-plane phototransistor with an out-of-plane vertical MoS_2_ p–n junction as a sensitizing scheme. The vertical built-in field is introduced for the first time in the transport channel of MoS_2_ phototransistors by facile chemical surface doping, which separates the photo-excited carriers efficiently and produces a photoconductive gain of >10^5^ electrons per photon, external quantum efficiency greater than 10%, responsivity of 7 × 10^4^ A W^−1^, and a time response on the order of tens of ms. This taken together with a very low noise power density yields a record sensitivity with specific detectivity $D^*$ of 3.5 × 10^14^ Jones in the visible and a broadband response up to 1000 nm.

## Abstract (web summary)

Photodetectors based on 2D transition metal dichalcogenides exhibit ever increasingly competitive performance, yet not superior to that of alternative technologies. Here, the authors devise a MoS_2_-based phototransistor with an out-of-plane junction, yielding a record detectivity combined with broadband response.

## Introduction

Two-dimensional (2D) transition metal dichalcogenides (TMDs) have emerged as a new optoelectronic platform with the promise to enable optoelectronic functionalities^1–11^ in the 2D form factor leveraging some unique properties, including their high optical absorption coefficient, favorable direct band gap, high carrier mobility^12–14^, mechanical flexibility, and potential for large scale growth and processing^15, 16^. So far, many efforts have been made to develop TMDs 2D-based photodetectors with ultrafast response^17^ and high responsivity^11^, with a view to enable their widespread application in remote sensing, camera imaging and optical communications. TMDs-based photodiodes with p–n junctions have been fabricated with transferred van der Waals heterostructures^18^ or chemical vapor deposition grown hybrids^19^; however, they are also characterized by low responsivity due to the absence of a photo-gain mechanism or by persistent photoconductivity which is unsuitable for photodetector applications. Until now photodetectors based entirely on 2D-TMDs have not outperformed other established technologies and the reported sensitivities have thus far been below that of alternative hybrid^20–23^ or standard silicon photodetectors. Reaching compelling sensitivity in 2D-TMDs photodetectors has been impeded by the absence of an intrinsic sensitization mechanism.

Here, we present an all-2D-based photodetector that consists of an out-of-plane (vertical) p–n MoS_2_ homojunction that acts as the photo-sensitizing layer of the underlying n-type MoS_2_ transistor. The internal built-in field created from the p–n junction facilitates the photo-excited carrier separation and leads to a significant photo-gating effect. As a result this hybrid detector yields a responsivity of 7 × 10^4^ A W^−1^ and a record measured detectivity of 3.5 × 10^14^ Jones with a time response on the order of 10 ms demonstrating the highest sensitivity of 2D-based photodetector to date^11, 24–35^.

## Results

### Device scheme

**Fig. 1:**
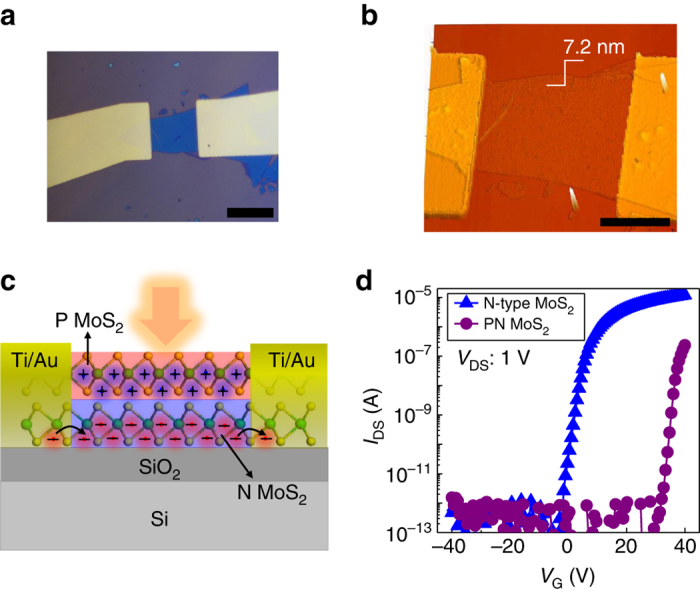
MoS_2_-based phototransistors with an out-of-plane PN homojunction. **a** Optical microscopy image of the few layer MoS_2_-based device with Ti/Au as source and drain electrodes, the *scale bar* is 20 µm. **b** Atomic force microscopy image of the device depicting a thickness of 7.2 nm. The *scale bar* is 10 µm. **c** Schematic diagram of the device after surface doping under light illumination consisting of P-MoS_2_ on top surface and N-MoS_2_ underneath. The photo-excited holes remain trapped in P-MoS_2_ while the electrons transport and recirculate in N-MoS_2_ channel. **d** Transfer characteristics of the pure N-MoS_2_ detector and surface doped device with an out-of-plane PN junction, showing the *V*_T_ shift towards positive-gate voltage

The few layer MoS_2_ flakes were exfoliated on the SiO_2_/Si substrate using the micromechanical exfoliation method. The Ti (2 nm)/Au (50 nm) electrodes were then fabricated by E-beam lithography and metal evaporation technique. After annealing the devices under N_2_ atmosphere at 150 °C for 2 h to improve the contact quality, the electrical and photosensitive properties were then measured before and after surface doping at room temperature and ambient environment. The optical microscope and atomic force microscopy images of the device are shown in Fig. 1a, b, respectively. The thickness of the device is measured 7.2 nm, suggestive of a 10-layer thick MoS_2_ channel. To form the MoS_2_ PN junction in the out-of-plane (vertical) direction of the device channel (Fig. 1c), we employed electronic doping. P-type doping techniques for MoS_2_ have been reported recently including plasma treatment^36, 37^, niobium physically doping^38, 39^, and gold chloride (AuCl_3_) chemically doping^40–42^. Among them, AuCl_3_ doping is one of the most facile approaches for P-type doping of MoS_2_ and has therefore been employed in this work. Here, we used low-concentrated AuCl_3_ solutions as P dopants to contain the P-type doping of MoS_2_ on the top layers of the few layer MoS_2_ channel. Optimization of the amount of doping has been executed and a concise summary of the progressive doping towards a totally p-type MoS_2_ channel is shown in the Supplementary Fig. 1. From the transfer characteristics under dark before and after doping as shown in Fig. 1d, the threshold voltage (*V*_T_) shifts dramatically from −3 V to 30 V due to the depletion of charges across the PN junction in which electrons from N-MoS_2_ are compensated by the holes of the P-MoS_2_, to equilibrate the Fermi level, forming a depletion region and essentially result in a more intrinsic MoS_2_ channel (Fig. 1d). The effective carrier density calculated from the transfer characteristics drops from 5.6 × 10^17^ cm^−3^ to 2.8 × 10^14^ cm^−3^ after the formation of the PN MoS_2_ transistor channel. The calculated mobility after the doping process is slightly reduced from 47 cm^2^ V^−1^ s^−1^ to 13.5 cm^2^ V^−1^ s^−1^, likely due to increased impurity scattering, yet it still remains high enough to serve the purpose of a highly performing phototransistor.

### Operation mechanism and responsivity

**Fig. 2:**
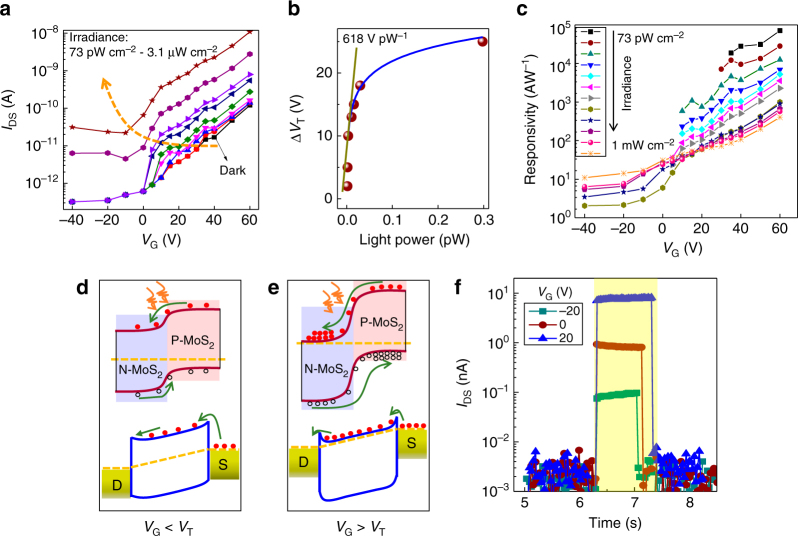
Underlying mechanism of photodetection in the detector. **a** Back-gate dependence of drain current under dark and different irradiance intensities, the *orange dash arrow* shows the *V*_T_ shift towards negative gate voltage with increasing illumination intensity due to the photo-gating effect. **b** *V*_T_ shift (∆*V*_T_) as a function of light power on the detector, the *blue line* is the non-linear fitting at relatively high light power regime and the *dark yellow line* shows a slope as high as 618 V pW^−1^ at low light power. **c** Responsivity of the device as a function of back gate at *V*_DS_ of 10 V and under varying irradiance, demonstrating the increased responsivity and more sensitive detection capability by applying large-positive gate voltage. **d** Energy band diagram of the out-of-plane PN junction at the interface (*top panel*) and the N-MoS_2_ transport channel (*bottom panel*) with applied bias under *V*_G_ < *V*_T_. **e** The same with **d** but under *V*_G_ > *V*_T_ showing the more efficient charge separation and recirculation due to the larger built-in field and negligible contact barrier. **f** Temporal response of the device at different back-gate voltage and *V*_DS_ of 10 V and under irradiance of 31.7 µW cm^−2^

Now we turn to the underlying mechanism of photodetection in the PN junction-sensitized MoS_2_ photodetector. In this device, the bottom N-MoS_2_ serves as the carrier transport channel while the top P-MoS_2_ is effectively isolated from the metal contacts due to the large Schottky barrier^37^. Upon light illumination, photo-generated carries in the PN junction are spatially separated under the internal built-in field of the junction with holes transferred to the P-type region and electrons populating the N-type MoS_2_ channel (Fig. 1c). This acts effectively as a photo-gating effect where the presence of these charges modulates the conductance of the N-MoS_2_ channel. By applying drain source bias *V*_DS_, photo-generated electrons drift and recirculate in the N-MoS_2_ channel before they recombine with the holes present in the P-MoS_2_. The recombination is retarded due to the formation of the built-in field across the out-of-plane PN junction giving rise to the possibility of photoconductive gain as the ratio of the carrier lifetime *τ*_life_ over the transit time *τ*_transit_. Figure 2a shows the back-gate dependence of source drain current under dark and light illumination at different intensities. *V*_T_ shifts gradually from 30 V to −10 V with increasing light intensity due to the accumulation of photo-generated electrons in the N-MoS_2_ channel under illumination. The *V*_T_ change (∆*V*_T_) induced by the photo-gating effect is plotted in Fig. 2b and reaches a value as high as 618 V pW^−1^. The ∆*V*_T_ shift provides a direct measure of the external quantum efficiency (EQE) given as the number of photo-generated charges transferred to the N-type MoS_2_ channel per single incident photon. The equation that describes this is given as:

1 $${\rm{EQE}} = \frac{1}{q}\frac{{\partial Q}}{{\partial n}} = \frac{1}{q}C\frac{{\partial P}}{{\partial n}}\frac{{\partial {V_{\rm{T}}}}}{{\partial P}}$$

where *Q* is the number of photo-induced charges; *q* is the elementary charge; *n* is the number of incident photons per second; *P* is the incident optical power; *C* is the capacitance which can be given by equation *C* = *ε*_0_ *ε*_r_ *A*/*d*, where *ε*_0_ is vacuum dielectric constant, *ε*_r_ (3.9) and *d* (285 nm) are dielectric constant and thickness of SiO_2_, respectively, *A* is active area of the detector; $\partial P = \tau \partial n\frac{{hc}}{\lambda }$, where *λ* is the wavelength, *h* is the Planck’s constant, *c* is the speed of light, and *τ* is the time constant of the detector; $\frac{{\partial {V_{\rm{T}}}}}{{\partial P}}$ here is 618 V pW^−1^. Based on this calculation the EQE is estimated at ~10% at 635 nm for an experimentally measured time constant of 50 ms at the same experimental conditions that the detector has been characterized. This EQE value is several orders of magnitude higher than prior reports in 2D semiconductors^6, 8, 43–46^ and it is rather remarkable considering a photodetector thickness of only 7 nm thanks to the very high absorption of the 2D-TMD active layer.

The electrical response of the detector upon illumination is determined by its responsivity defined as:

2 $$R = \frac{{{I_{{\rm{ph}}}}}}{{PS}}$$

in units of A W^−1^, where *I*_ph_ is the photocurrent; *P* is the incident light power density and *S* is the active area. Figure 2c shows the responsivity dependence of the detector on the backgate voltage at various optical intensities reaching a value as high as 7 × 10^4^ A W^−1^ for the lowest incident detectable power of the detector at 0.25 fW. The detector operates most sensitively at a back-gate voltage in the range of 40–60 V which is close to the maximum transconductance point. The dependence of responsivity on the backgate bias can be explained from the schematic diagram of the energy band of the device under negative and positive gate with the applied bias shown in Fig. 2d, e, respectively. Due to the charge screening effect of the N-MoS_2_ underneath^47^, the back-gate modulates mainly the Fermi level in the N-MoS_2_ channel, leaving the top P-MoS_2_ layer unaffected. At negative gate bias, the N-MoS_2_ is depleted with the Fermi level falling at the middle of the band gap. Thus the built-in field of the out-of-plane PN junction is reduced due to the lack of electrons in the N-MoS_2_ to compensate the holes of the P-MoS_2_. This leads to a low built-in field, essential for efficient charge separation (*top panel* in Fig. 2d) as well as a large Schottky barrier contact between the drain source electrodes and the N-MoS_2_ channel that further hinders charge recirculation and therefore gain (*bottom panel* in Fig. 2d). On the other hand, the built-in field increases by applying a positive gate bias with the Fermi level in the N-MoS_2_ moving towards the conduction band. This facilitates a more efficient charge separation across the PN junction (*top panel* in Fig. 2e) as well as the possibility of gain in view of the formation of Ohmic contacts of the N-MoS_2_ channel with the drain and source electrodes (*bottom panel* in Fig. 2e). The gate-modulated Fermi level and effective contact barrier height is consistent with previous reports^11, 48^, the decreased effective contact barrier by increasing the back gate is due to the reduced depletion width (i.e., sharp band bending near the contact) that improves the thermionic emission or thermally assisted tunneling process. The significant charge transport reported in the out-of-plane direction of MoS_2_^49, 50^ also supports the effective charge separation and transfer under the built-in field in the out-of-plane PN junction reported herein.

To confirm the above mechanism and to ensure that the transistor channel is governed by the N-type MoS_2_ whereas the P-type MoS_2_ serves as a sensitizing layer, we have also implemented an insulating Al_2_O_3_ window layer to prevent the p-type doping of the MoS_2_ near the contacts and ensure that the source and drain electrodes are in contact solely with the N-type MoS_2_ channel. These detectors performed equally well with the ones reported in the main text (Supplementary Fig. 2). Further doping of the devices with and without Al_2_O_3_ window was also carried out to heavily dope the MoS_2_ channel and eventually collapse the formation of the PN junction. Responsivity and sensitivity deteriorated significantly upon this (Supplementary Figs. 3, 4) due to the absence of the photo-sensitizing scheme in this case, which further confirms the important contribution of the out-of-plane MoS_2_ PN junction to the high performance reported.

### Temporal response and sensitivity

**Fig. 3:**
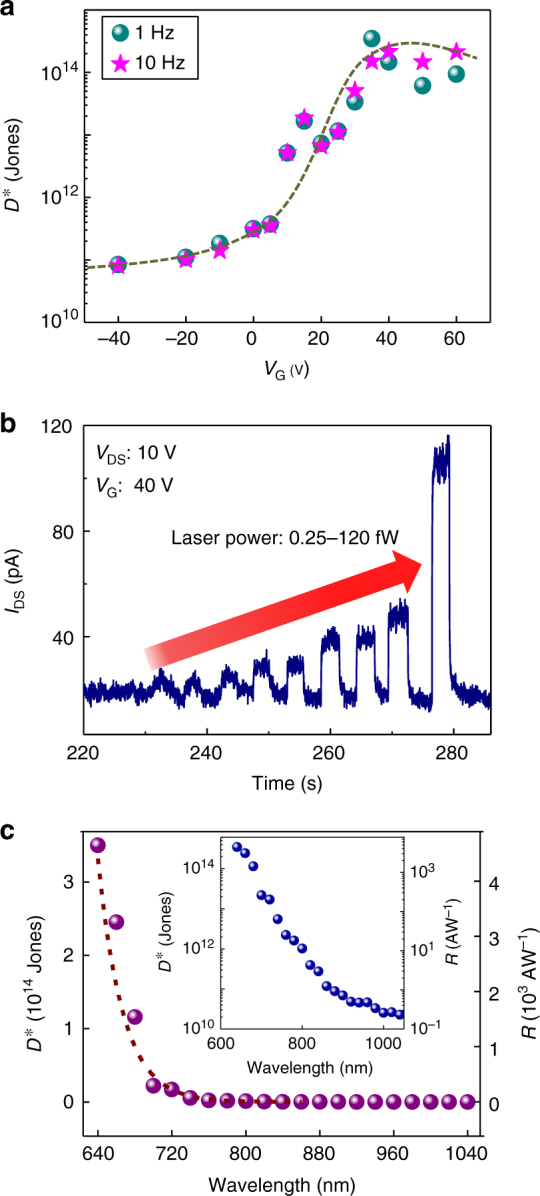
Ultrahigh sensitivity and spectrum. **a** Specific detectivity $D^*$ as a function of back-gate voltage at *V*_DS_ of 10 V and optical modulated frequency of 1 Hz and 10 Hz, the *dash line* is a guide to the eye. **b** Time dependence of photo-response with increasing irradiance showing a direct measurement of laser power as low as 0.25 fW. **c** Spectral detectivity and responsivity under irradiance of 1 nW cm^−2^ and at *V*_DS_ of 10 V and *V*_G_ of 35 V, the insert is the spectrum with log scale for *y*-axis showing the extended spectrum to near-infrared region (1000 nm) with high sensitivity

The temporal photo-response of the detector is measured under different back-gate values (Fig. 2f), yielding a relaxation timescale of 10 ms, significantly faster than that of pure MoS_2_ photodetectors (Supplementary Fig. 5 and refs. ^11, 25^) at irradiance of tens of μW cm^−2^. To determine the gain of the detector defined as:

3 $$G = \frac{{{\tau _{{\rm{life}}}}}}{{{\tau _{{\rm{transit}}}}}}$$

we first consider the *τ*_transit_, which is inversely proportional to the electron mobility and can be defined as ${\tau _{{\rm{transit}}}} = \frac{{{L^2}}}{{\mu {V_{{\rm{DS}}}}}}$, where *L* is length of the channel, *µ* is the mobility and *V*_DS_ is the applied bias; thus the transit time is calculated to be ~32 ns. From the temporal response of the sensitized detectors at much lower irradiance levels (shown in Fig. 3b), the carrier lifetime is estimated on the order of 50 ms. As a result, multiple electrons are recirculated in the MoS_2_ channel following a single electron–hole photo-generation, leading to a photoconductive gain on the order of 10^5^. This allows us then to estimate the EQE of the detector from the formula:

4 $$R = \frac{{\lambda q}}{{hc}} \times {\rm{EQE}} \times G$$

where *λ* is the wavelength, *q* is the elementary charge, *h* is the Planck’s constant, and *c* is the speed of light. Based on this, the EQE of the detector is calculated at 9% in very good agreement with the calculation of EQE based on the ∆*V*_T_ shift.

Besides the high responsivity recorded, another key feature for ultrasensitive photodetection is the low noise current floor. The details of the noise analysis of the photodetector is summarized in Supplementary Fig. 6. Briefly, with applying a constant drain bias and back gate, the time-resolved dark current is measured. By taking the Fourier transform of dark current traces, the noise power densities are obtained as shown in Supplementary Fig. 6b, then the noise spectral densities *S*_n_ are extracted at frequencies of 1 Hz and 10 Hz under exactly the same conditions as the optical measurements were performed (same *V*_G_ and *V*_DS_), resulting in noise values between 0.1 pA Hz^−1/2^and 1.4 pA Hz^−1/2^ in the whole back-gate range (Supplementary Fig. 6c). To experimentally assess the sensitivity of the detector, we measured the specific detectivity $D^*$ which is defined as:

5 $${D^{\rm{*}}} = \frac{{\sqrt {AB} }}{{{\rm{NEP}}}} = \frac{{R\sqrt A }}{{{S_{\rm{n}}}}},$$

in units of cm Hz^1/2^ W^−1^ (or Jones), where NEP is the noise equivalent power in W, *R* is the responsivity in A W^−1^, *A* is the active area of the detector in cm^2^, *B* is the noise bandwidth in Hz, and *S*_n_ is the noise current spectral density of the detector in A Hz^−1/2^. Figure 3a illustrates the $D^*$ as function of the back-gate voltage. $D^*$ increases with increasing backgate to reach its maximum value at the point which the maximum transconductance of the N-type MoS_2_ transistor coincides with the maximum EQE of the detector and the preservation of low-dark current. In this regime the $D^*$ reaches a value range of 1 × 10^13^ Jones to 3.5 × 10^14^ Jones at modulation frequencies of 1 Hz and 10 Hz, respectively, the highest recorded to date for any 2D-based photodetector. This high $D^*$ results from the highly efficient charge separation, large photoconductive gain as well as the very low noise spectral density in this class of 2D-only based photodetectors. The reported $D^*$ is higher by at least two orders of magnitude over prior reports of 2D materials based photodetectors^11, 24–35^ and on par with bulky silicon photomultiplier detectors^51^. Thanks to the high sensitivity of the detector we have directly measured optical power as low as 0.25 fW using an source-measure unit (SMU)-based semiconductor parameter analyser (Fig. 3b) which determines the noise equivalent power (NEP) of the detector in broad bandwidth conditions that is compatible with and used in standard complementary metal–oxide–semiconductor (CMOS) readout electronics. Considering an integrated noise bandwidth of 50 Hz that corresponds to the sampling rate of 20 ms, the $D^*$ is found to be ~5.2 × 10^13^ Jones, the highest ever reported and directly measured under broad bandwidth conditions in any class of 2D-based photodetector. The reported photodetector exhibits a $D^*$ greater than 10^13^ Jones for wavelengths up to 700 nm. Due to the multilayer nature of the MoS_2_ selected as the transport channel and absorption layer, the optical spectral coverage of our detectors is extended from visible to near-infrared region (Fig. 3cc) in agreement with the band gap of ~1.2 eV of multilayer MoS_2_^1, 12^.

## Discussion

This work reports an ultrasensitive detector comprising solely 2D-TMDs materials enabled by band engineering in the out-of-plane direction through doping. It is noted that the optimized thickness of MoS_2_ in this work was found to be 7–11 nm (see details in Supplementary Figs. 7–9). The formation of the out-of-plane PN junction serves as a novel sensitizing mechanism for 2D-TMDs phototransistors and paves the way towards the use of the concept in other 2D semiconductors or in combination of those to facilitate sensitization, particularly those possessing low band gap to extend the spectral coverage of the 2D materials realm.

## Methods

### Device fabrication

The MoS_2_ crystals were purchased from the 2D semiconductors corporation. The few layer MoS_2_ was then exfoliated with PDMS tape on Si/SiO_2_ (285 nm) wafer using the micromechanical exfoliation method. Before the device fabrication, the substrate with MoS_2_ on top was soaked into acetone for 2 h at 60 °C to remove the residual glue. Metal contacts were then fabricated by the laser writing lithography, and Ti (2 nm) and Au (50 nm) electrodes were evaporated by e-beam and thermal evaporation, respectively. Finally, the devices were annealed at 150 °C for 2 h under high purity of N_2_ atmosphere to improve the contact quality. For the devices with Al_2_O_3_ window, both electrodes were covered by insulating Al_2_O_3_ with atomic layer deposition technique (ALD) (Savannah 200, Cambridge Nanotech). The ALD process was performed in 300 cycles leading to ~30 nm of oxide thickness.

### AuCl_3_ solution preparation and surface doping

The synthesis of AuCl_3_ dopant is according to the previous reports^40–42^. Briefly, 30 mg gold chloride (AuCl_3_) powders (≥99.99 %, Sigma Aldrich) were dissolved in the 5 ml nitromethane (≥95 %, Sigma Aldrich), then the solutions (20 mM) were sonicated for 3 h at 60 degrees followed by filtration to filter the large Au aggregates. The 20 mM AuCl_3_ solutions were then diluted with different concentration (10 mM and 5 mM). Note that all operations were performed in glove box to protect the reagents from the air environment. About 3–5 drops of 5 mM AuCl_3_ solutions were dropped on the SiO_2_/Si substrate with MoS_2_ devices on top and spin-coated at 3000 rpm for 1 min, then baked on the hotplate at 100 °C for 5 min.

### Device characterization

All the measurements were performed in ambient conditions using an Agilent B1500A semiconducting device analyzer. For spectral photo-response measurements the devices were illuminated with fiber-coupled and spectrally filtered light from a supercontinuum light source (SuperKExtreme EXW-4, NKT Photonics). Responsivity and temporal response times were measured under short-pulsed light at a wavelength of 635 nm from a four-channel laser controlled with an Agilent A33220A waveform generator. Several dark current traces were measured with the Agilent system (Agilent B1500A) under exactly the same conditions as the optical measurements were performed (same *V*_G_ and *V*_DS_) at a sampling rate of 50 Hz. We obtained the noise spectral density by calculating the Fourier transformation of dark current traces.

### Data availability

The data that support the findings of this study are available from the corresponding authors upon request.

## Electronic supplementary material

Supplementary Information
