# Supplementary material for: Ultrasensitive all-2D MoS2 phototransistors enabled by an out-of-plane MoS2 PN homojunction
Source: Nat Commun. 2017 Sep 18;8:572. doi: 10.1038/s41467-017-00722-1 (PMC5603552; doi:10.1038/s41467-017-00722-1)
Supplement: Supplementary file 1 — Supplementary Information [file 41467_2017_722_MOESM1_ESM.pdf]

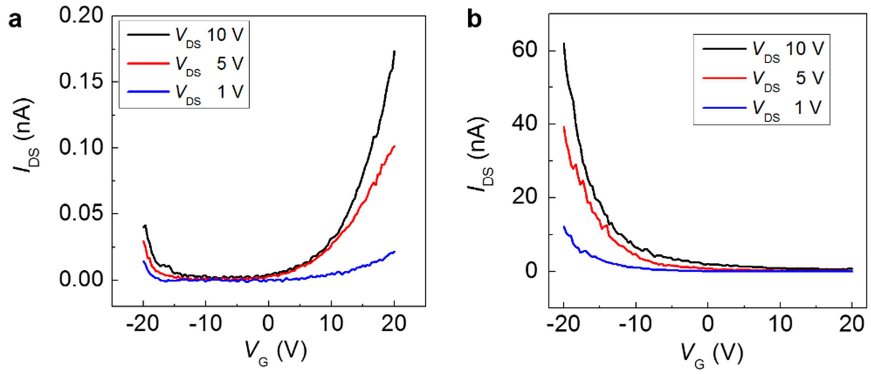

Supplementary Figure 1. MoS_2 Phototransistors with further surface doping. (**a**) Transfer characteristics of the same detectors discussed in main text with 5 mM $AuCl_3$ solution doped for the second time at V_{DS} of 1 V, 5 V and 10 V, showing the ambipolar behaviour. This indicates that the bottom N-MoS_2 channel is deeply depleted from the major P doped MoS_2 on top. (**b**) Transfer characteristics of the detectors with 10 mM $AuCl_3$ solution doped for the third time at V_{DS} of 1 V, 5 V and 10 V, showing the completely P-Type behaviour. This indicates that the whole N-type MoS_2 layer has been P doped.

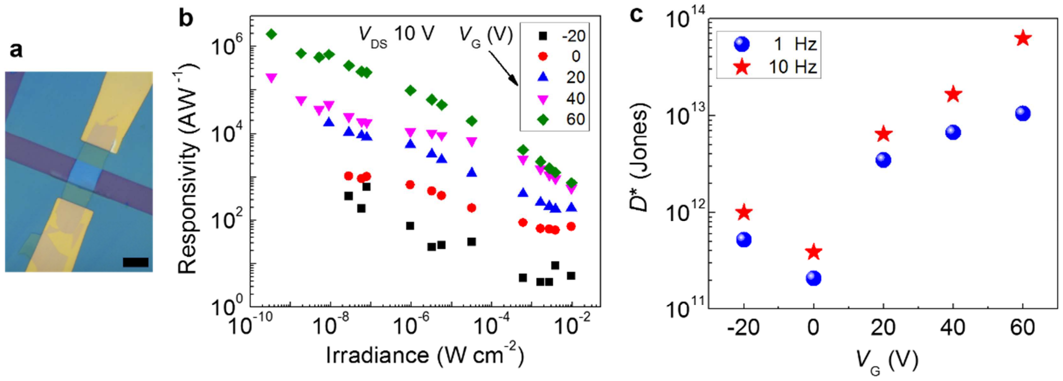

Supplementary Figure 2. Phototransistors with insulating Al_2O_3 window covering both electrodes and with same surface doping. (**a**) Optical microscopy image of few layer MoS_2 based detectors with Al_2O_3 window (40 nm). The scale bar is 10 µm. (**b**) Responsivity at different back gate voltage and V_{DS} of 10 V, as a function of light power density. The responsivity trends with different back gate and varying light illumination intensity are very similar with that in the devices without Al_2O_3 window as discussed in text. The R here can reach 10^6 A W^{-1} at low irradiance and high back gate, which can come from the efficient charge separation and high photoconductive gain from the high mobility of N-MoS_2 transport channel. (**c**) Back gate voltage dependence of specific D^* at V_{DS} of 10 V and light modulated frequency of 1 Hz and 10 Hz, which also ranges from 1×10^{12} to 1×10^{14} Jones. The equally photodetection performances in this phototransistor with Al_2O_3 window compared to the device without window indicate that the bottom N-MoS_2 plays a main role in charge transport and recirculation, while the top P-MoS_2 acts as the sensitizing layer forming an out-of-plane PN junction at interface and facilitating the carrier generation and separation.

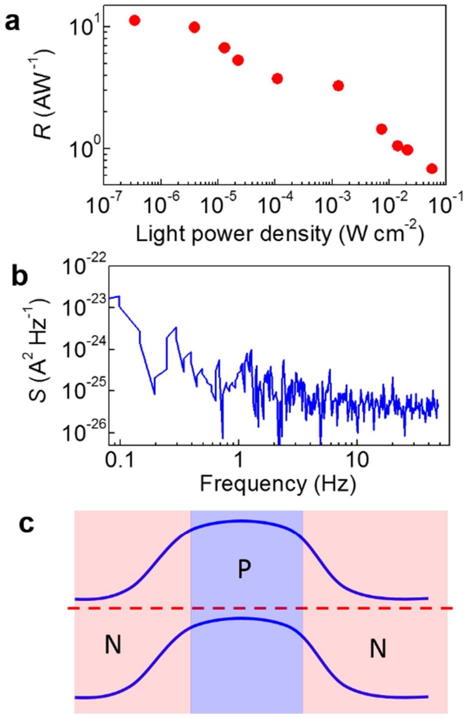

Supplementary Figure 3. Performance of the further doped MoS_2 phototransistor with Al_2O_3 window. (**a**) Responsivity as a function of incident light power density at V_{DS} of 10 V and V_G of 20 V. (**b**) Noise power density of the device at the same measurement conditions. (**c**) Schematic diagram of energy band of heavily doped systems where uncovered MoS_2 is P-type while covered MoS_2 is N-type. Thus two opposite lateral PN junctions are formed, and one of the junction can form large barrier for the photo-excited carrier. The responsivity is 10 A W^{-1} under low irradiance and the specific D^* is 5×10^{10} Jones at noise frequency of 1 Hz which are several orders of magnitude smaller than the main device. The much drop in performance is due to the heavily doping of N-MoS_2 channel and absence of out-of-plane PN junction.

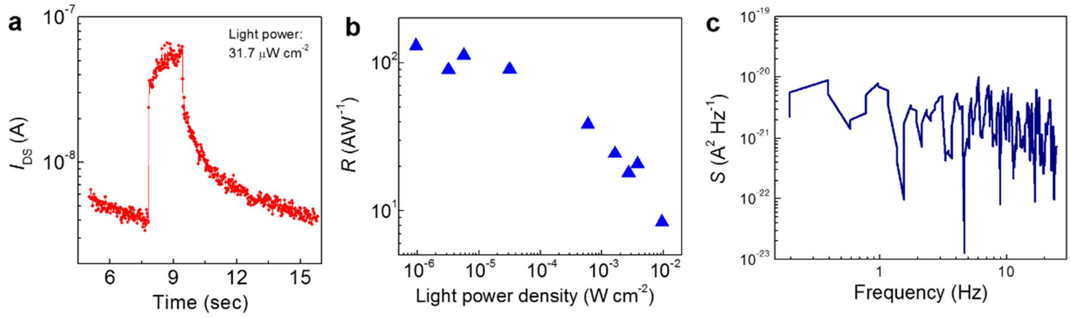

Supplementary Figure 4. Performance of the further doped MoS_2 phototransistor without Al_2O_3 window. (**a**) Temporal response of the device at V_{DS} of 10 V and V_G of 20 V. (**b**) Responsivity as a function of light power density. (**c**) Noise power density of deeply doped systems at the same measurement conditions. The photodetection performance for the deeply doped systems is also much dropped with longer decay time of ~1 s, smaller responsivity of 100 A W^{-1} and sensitivity of 3×10^9 Jones, which can also be attributed to the removal of the out-of-plane PN junction and less photo-gain production.

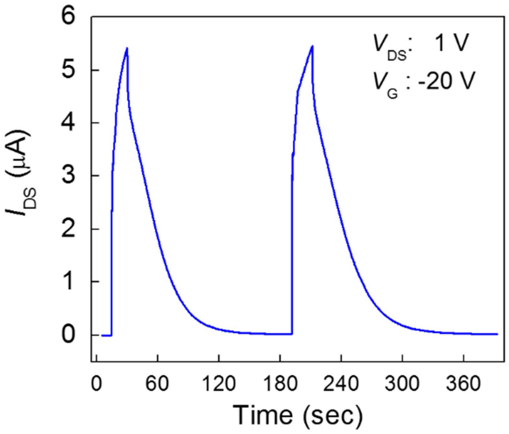

Supplementary Figure 5. Temporal response of pristine MoS_2 device. Photo-response of the few layer MoS_2 based phototransistors before the surface doping at V_{DS} of 1 V and V_G of -20 V, showing the very slow response due to the numerous defects or adsorbates in MoS_2.

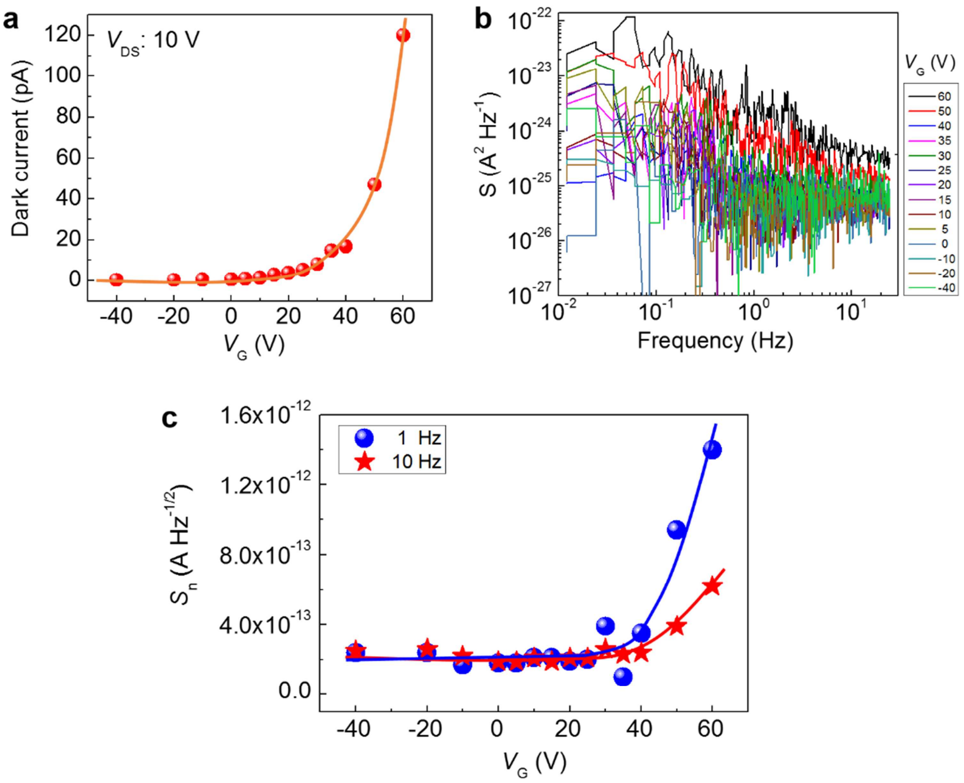

Supplementary Figure 6. Dark current and noise analysis of the detectors with out-of-plane PN junction. The time resolved dark current traces were measured with Agilent system (Agilent B1500A) under different back gate voltage and V_{DS} of 10 V with exactly the same conditions as the optical measurements were performed at a sampling rate of 50 Hz. (**a**) The extracted dark current value from the dark current traces as a function of applied V_G. The dark current can remain very low value ranging from 1-120 pA even at high gate voltage. (**b**) By calculating the Fourier transformation of dark current traces, we obtained very low noise power density in the whole range of applied gate and the extracted noise spectral density S_n is ranged between 1×10^{-13} A Hz$^{-1/2}$ and 1.4×10^{-12} A Hz$^{-1/2}$ at the noise frequency of 1 Hz and 10 Hz, plotted in (**c**) showing the very low noise spectral density which can enable the ultrahigh sensitivity of the detector.

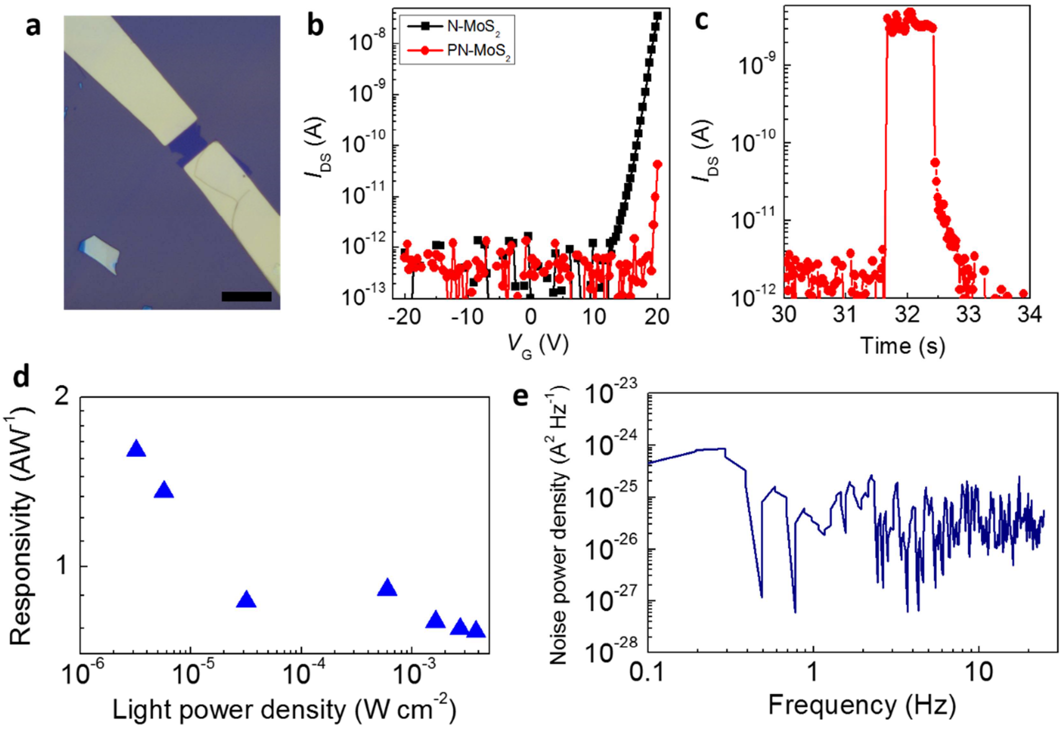

Supplementary Figure 7. Performance of the doped bilayer MoS_2. (**a**) Optical microscopy image of bilayer MoS_2 with a thickness of ~ 1.3 nm based phototransistors. The scale bar is 10 μm. (**b**) Transfer characteristics of bilayer MoS_2 before and after doping with 5 mM $AuCl_3$. The threshold voltage shifts towards to positive gate due to the p-type doping and the current is much reduced due to the electrons depletion. Based on the calculation, the pristine N-MoS_2 has a mobility of 5 cm^2 V^{-1} s^{-1}, after doping the mobility is much dropped to only 1.2 × 10^{-2} cm^2 V^{-1} s^{-1}. (**c**) Temporal response of the doped device under 9.5 mW cm^{-2} light illumination showing a fast response of ~20 ms. (**d**) Responsivity as a function of incident light power density at V_{DS} of 10 V and V_G of 20 V, showing the low responsivity below 2 A W^{-1}. (**e**) Noise power density of the device by calculating the Fourier transformation of dark current traces under exactly the same conditions as the optical measurements were performed (same V_G and V_{DS}) at a sampling rate of 50 Hz.

Based on the noise power density, the specific D^* at 1 Hz is calculated to be 8.7 × 10^9 Jones which is around 4 orders of magnitude smaller than that in our main devices discussed

in main text. Due to the ultrathin feature of bilayer MoS_2, the same p-doping can lead to fully depletion of N-MoS_2. This results in the much dropped mobility and also the absence of out-of-plane PN junction. Thus less gain can be introduced in the bilayer MoS_2 phototransistors leading to poor responsivity and sensitivity.

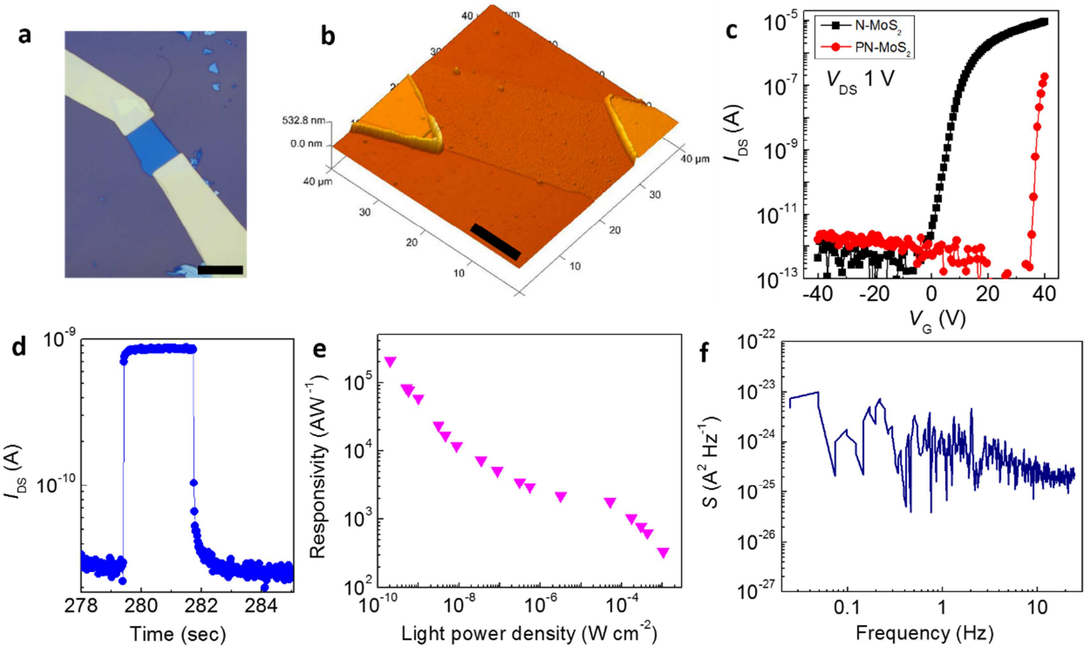

Supplementary Figure 8. Performance of the doped multilayer MoS_2 with 11.5 nm thickness. (**a**) Optical microscopy image and (**b**) Atomic force microscopy image of the multilayer MoS_2 phototransistors depicting a thickness of 11.5 nm corresponding to 17 layers. The scale bar is 20 μm in (a) and 10 μm in (b). (**c**) Transfer characteristics of the device before and after doping with 5 mM $AuCl_3$. The threshold voltage also shifts towards to positive gate due to the p-type doping. (**d**) Temporal response of the device under 3 μW cm^{-2} light illumination showing a fast response of ~20 ms at V_{DS} of 10 V and V_G of 20 V. (**e**) Responsivity as a function of incident light power density at V_{DS} of 10 V and V_G of 20 V, the responsivity can reach as high as 2×10^5 A W^{-1} under low irradiation. (**f**) Noise power density of the device, which can be used to calculate the specific D^* of 3.4×10^{14} Jones at 1 Hz.

Based on the calculation, the pristine multilayer MoS_2 has a mobility of 53.3 cm^2 V^{-1} s^{-1}, after doping the mobility is slightly decreased to 12.3 cm^2 V^{-1} s^{-1}. This device with 11.5 nm thickness exhibits an equal performance (*i.e.* fast response, high gain and sensitivity) compared to the main device with 7.2 nm thickness using the same doping level (5 mM $AuCl_3$).

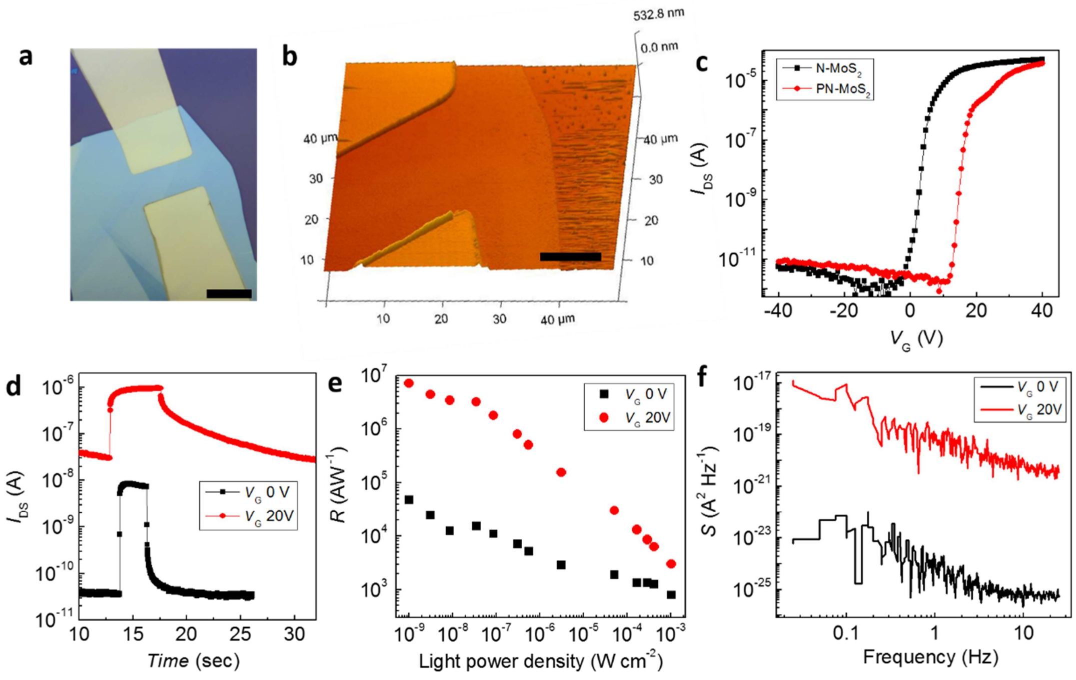

Supplementary Figure 9. Performance of the doped multilayer MoS_2 with 18.3 nm thickness. (**a**) Optical microscopy image and (**b**) Atomic force microscopy image of thicker MoS_2 phototransistors showing a thickness of 18.3 nm corresponding to 28 layers. The scale bar is 20 μm in (a) and 10 μm in (b). (**c**) Transfer characteristics of the device before and after doping with 5 mM $AuCl_3$. The threshold voltage slightly shifts towards to positive gate. (**d**) Temporal response of the device at V_G of 0 V and 20 V showing a slower response. Especially at V_G of 20 V, the decay time reached few seconds which presents intrinsic property of pure MoS_2. (**e**) Responsivity as a function of incident light power density at V_{DS} of 10 V and V_G of 0 V and 20 V. (**f**) Noise power density of the device measured at the same experimental conditions.

For this device, the mobility is 150 cm^2 V^{-1} s^{-1} and 123 cm^2 V^{-1} s^{-1} before and after doping, respectively. The slight change in transfer curves and mobility indicates that the doping influence on this device is less than that on other devices with thinner MoS_2 flakes. Because of the large thickness (~18.3 nm), the bottom MoS_2 layers may not be influenced by the P

dopants. However, an out-of-plane PN homojunction can still form in top layers which can produce large photo-gain resulting in a very high responsivity of 7×10^6 A W^{-1} and sensitivity of 5.3×10^{13} Jones at V_G of 20 V. The higher responsivity can be attributed to the higher photo-gain from larger mobility and longer carrier lifetime. The sensitivity is slightly dropped due to the significant noise at V_G of 20 V. Although we get high performance in this device, the slow response in such thick MoS_2 would limit its application. It can be predicted that the doping effect would be less and less with further increased thickness, and the pure MoS_2 itself can mainly contribute to the device performance. Thus the optimized thickness of MoS_2 with such doping level (5 mM $AuCl_3$) for best performance could be around 7-11 nm.
